# Address

**Published:** 1864-05

**Authors:** E. W. Hadley

**Affiliations:** President


### ADDRESS

To the first Dental Society of Chicago, by the President, E. W. Hadley. Delivered March 14, 1864.

*Gentlemen of the Chicago Dental Society:*

In assuming, for the first time, the discharge of my duties as presiding officer of your Society—the first regular association of Dentists in this great metropolis of the North-West, I deem it proper and legitimate to submit a few initial remarks bearing on the object and benefit of societies and organizations like ours, both to members and communities at large.

1st. The object of all combined effort, whether physical or mental, is, and should be, to ensure more complete and perfect results in any department of physics or science, to which each individual of the association is devoting his personal energies.

Thus, to use a masonic figure, while one brings stone, another brick, a third mortar, well tempered, and others lay the wall by the line and plummet, the temple of improvement will rise successfully, till the topmost stone is brought forth with shouting.

But, to drop the figure, let us inquire how to accomplish the most good, and attain the highest object of our association. I here remark interrogatively, is it not by the interchange of kindly thought? Courtesy of manners one to another? Carefully avoiding all jealousy in feeling or action, and cultivating the better powers of our being? Such are gentleness, goodness, forbearance, and charity; and allow me to say, "*the greatest of these is charity.*" This pre-eminent virtue will surely cover a multitude of our follies and faults.

In the details of our common calling, we shall naturally, *nay*, inevitably differ. But in all kindness, let not this difference alienate or interrupt the sincere wish that *all* should succeed, to that degree, at least, which shall entitle them to honorable positions among the fraternity of our profession.

With commendable pride we all aim at individual excellence. This is right; but as no *one* can monopolize a profession, let this individual excellence honorably attained, be diffused in a spirit of genuine liberality through the profession, and the enlightened public will appreciate the efforts of us all to meet with combined skill their every want.

Again, the thorough and practised Dental Surgeon makes no secret of his modes of operating or manipulating, even to his patients. For well he knows that none but the ignorant can be influenced by professional mysteries. Let our communications with each other, as members of this Society, and with those of our profession who are not connected with our organization, be characterized by generosity, frankness, civility; in fine, with all consideration consistent with the true gentleman. This leads me naturally to the remark, that the influence which well conducted associations send abroad among the profession throughout the land, is by no means to be overlooked. The importance which we *know* belongs to our art, is more readily admitted by this union, and our brethren of other cities and towns see in us a determination to dignify the calling by mutual assistance to all honorable members in their efforts at progress.

In a word, the benefits of our Society may be made large by systematic *work*, in the diffusion of all we know of the art and profession of *Dentistry*.

Allow me, gentlemen, just here to quote from divine authority (but with all reverence), yet with singular aptness, "In watering others, we shall ourselves also be watered."

One word in regard to the permanence of such societies as we have now inaugurated. Americans are prone to build *only* for the present, leaving the future to take care of itself. The farmer of the West, for instance, "squats" on his quarter section—hastily rolls the logs together for a house—

chinks the walls with moss and mud—thatches the roof with poles and prairie grass—brings his family to this inhospitable shelter, with the apology, "it *will do for the present.*"

This, I grant, is in many cases the work of "necessity" which "knows no law;" but in others, he is too eager to reap a rich crop for market, and thus *realize* with the outlay of little or no capital.

Not so with us. The formation of a society—a tabernacle—a dwelling-place for the family of our profession, whose rooms and places must eventually be occupied by others, who will be called in the future to fill our posts at the chair and in the laboratory. In a work like this we surely can see many reasons for deliberation.

Let the foundation be laid broad and firm, and ere you add one stone to the superstructure, look well if this temple will meet the wants and bear the criticism of our profession when we are among the *departed.*

Patience, perseverance and time, will mature our plans, and make them permanent, if laid carefully and correct. "*Festina Lente,*" "Make haste slowly," should be our motto—for what is worth doing at all is worthy of being done well.

Five — ten — twenty, nay, forty years soon pass, and although the shortest of these periods appears long when before us, look *back* on the longest and it shrinks to less than a span.

Disowning any attempt at, or thought of, dictation, in regard to the future conduct of our Society, I have endeavored to give you in brief, a few suggestions on associations, their object, aim and end.

Springing from good motives—conducted with earnest efforts from all—the results before enumerated will surely follow.

The field of usefulness is large, both in your private practice, and in connection with the Society just formed. You may plow deep and sow broad. The appliances for the cultivation of the art you profess, are ample. The scientific knowledge and mechanical skill of the last quarter century, elaborated especially for your benefit, are before you, and with such a capital none should despair of ultimate success.

To confirm my last remark, take a hasty look back, you gentlemen who can clearly remember just twenty years, and let me ask you, what was dentistry even then? Look still another ten years into the past, and you will find ample cause for self-gratulation that *you commenced* the practice of *Dentistry* in the latter part of the nineteenth century; that you were spared the mortification of repeated failures, for the want of that very light and knowledge so brightly, so broadly beaming all around you at the present day.

And now, gentlemen, while I formally thank you for the honor conferred, in placing my name at the head, and making me your presiding officer for a short period, I can not conceal the fact, that no superior attainments in the profession entitle me to this distinction, and I accept it only as a tribute to seniority of age and occupancy of this immediate field of our labor.

But at that period so rough was the soil, I could scarce anticipate a subsistence, much less expect a golden harvest, either in honors or profits. With the limited means of improvement at hand, one could do little more than live, and you may be sure my case was not an exception.

Far from being discouraged, I struggled on in the face of many obstacles, with the hope that as our city improved, the profession would be encouraged, and take rank with kindred arts and sciences. As advancement in *all* arts surely follows prosperity in trade and commerce, so our profession gained steadily in importance, and met every want of the community with progressive skill and energy.

And now, after more than twenty years of toil in what has grown around me to the dignity and consequence of a *mighty city*, I find myself surrounded by a "*Dental Society*," whose individual attainments in the profession may challenge the world.

In conclusion, permit me to say, that while I promise to preside over your deliberations with what little ability I possess, I shall look with confidence to the earnest support and assistance of my professional brethren who have so unitedly placed me here and I most heartily add the wish and the hope, that this Society, so happily begun, may continue—"NOT FOR A DAY, BUT FOR ALL TIME."

